# The American Veterinary College

**Published:** 1884-07

**Authors:** 


The American Veterinary College.—The annual Announcement of the above Institution opens with the intelligence of the absorption into its organization of the Columbia Veterinary College of this city.

However unfortunate the failure of a young but already far-famed Institution may be, the result in this case cannot be considered as other than a source of satisfaction to the veterinary profession in this country, assuring it, as it does, the possession of a school which bids fair to rival the best establishment of its kind in Europe. It certainly cannot be surpassed in its facilities for affording opportunities to students to acquire practical knowledge of their profession in the numerous private and public stables and hospitals to be found in New York city.

The anatomical and pathological preparations which the College museum contains are superior to those of any institution of its kind in America.

The Hospital Department is an important factor in affording clinical instruction, two thousand five hundred animals having been entered in the record books during the past year. Apropos of this, we refer our readers to a letter from Dr. Liautard relating to the subscription plan. C.

